# Differential Expression of Viral Transcripts From Single-Cell RNA Sequencing of Moderate and Severe COVID-19 Patients and Its Implications for Case Severity

**DOI:** 10.3389/fmicb.2020.603509

**Published:** 2020-10-16

**Authors:** Teng Liu, Peilin Jia, Bingliang Fang, Zhongming Zhao

**Affiliations:** ^1^Center for Precision Health, School of Biomedical Informatics, The University of Texas Health Science Center at Houston, Houston, TX, United States; ^2^Department of Thoracic and Cardiovascular Surgery, The University of Texas MD Anderson Cancer Center, Houston, TX, United States; ^3^MD Anderson Cancer Center UTHealth Graduate School of Biomedical Sciences, Houston, TX, United States; ^4^Human Genetics Center, School of Public Health, The University of Texas Health Science Center at Houston, Houston, TX, United States

**Keywords:** COVID-19, SARS-CoV-2, virus evolution, cell infection rate, ORF10, virus gene fusion, viral fusion evolution, virulence

## Abstract

With steady increase of new COVID-19 cases around the world, especially in the United States, health care resources in areas with the disease outbreak are quickly exhausted by overwhelming numbers of COVID-19 patients. Therefore, strategies that can effectively and quickly predict the disease progression and stratify patients for appropriate health care arrangements are urgently needed. We explored the features and evolutionary difference of viral gene expression in the SARS-CoV-2 infected cells from the bronchoalveolar lavage fluids of patients with moderate and severe COVID-19 using both single cell and bulk tissue transcriptome data. We found SARS-CoV-2 sequences were detectable in 8 types of immune related cells, including macrophages, T cells, and NK cells. We first reported that the SARS-CoV-2 ORF10 gene was differentially expressed in the severe vs. moderate samples. Specifically, ORF10 was abundantly expressed in infected cells of severe cases, while it was barely detectable in the infected cells of moderate cases. Consequently, the expression ratio of ORF10 to nucleocapsid (N) was significantly higher in severe than moderate cases (*p* = 0.0062). Moreover, we found transcription regulatory sequences (TRSs) of the viral leader sequence-independent fusions with a 5’ joint point at position 1073 of SARS-CoV-2 genome were detected mainly in the patients with death outcome, suggesting its potential indication of clinical outcome. Finally, we identified the motifs in TRS of the viral leader sequence-dependent fusion events of SARS-CoV-2 and compared with that in SARS-CoV, suggesting its evolutionary trajectory. These results implicated potential roles and predictive features of viral transcripts in the pathogenesis of COVID-19 moderate and severe patients. Such features and evolutionary patterns require more data to validate in future.

## Introduction

The new coronavirus disease 2019 (COVID-19) has casted an imminent threat to people all over the world. COVID-19 is caused by a novel human-infecting betacoronavirus designated as severe acute respiratory syndrome coronavirus 2 (SARS-CoV-2). As of this manuscript updated writing (September 5, 2020), this life-threatening pneumonia pandemic has quickly caused more than 26.7 million confirmed cases and over 875,000 deaths globally since it first emerged in December 2019 (Sohrabi et al., 2020; Yang et al., 2020). SARS-CoV-2 genome, like SARS-CoV, is under frequent mutations which likely caused the change of infection rate (Zhao et al., 2004). As a positive-sense, single-stranded RNA virus with an approximate 30 kb in length of its genomic RNA (gRNA) (Pal et al., 2020), SARS-CoV-2 infects host cells through cell surface proteins ACE2 and TMPRSS2 (Hoffmann et al., 2020; Zhou et al., 2020). Inside host cells, SARS-CoV-2 gRNA can synthesize subgenomic RNAs (sgRNAs) during its replication (Khailany et al., 2020). The canonical coronaviral sgRNA contains a 5’ leader sequence with ∼65 nucleotides (nt) in length (Wu and Brian, 2010), which is fused to the “body” sequence to form the sgRNA with transcription regulatory sequences (TRSs) (Kim et al., 2020). A total of 10 open reading frames have been identified in SARS-CoV-2 gRNA, which generate multiple viral proteins including RNA-dependent RNA polymerase (ORF1ab), spike protein (S), ORF3a, envelope protein (E), membrane protein (M), ORF6, ORF7a, ORF8, nucleocapsid protein (N), and ORF10. Among them, S, E, M, and N are structural proteins that package virus gRNA to generate progeny (Lu et al., 2020; Wolfel et al., 2020). The ORF1ab gene encodes a polyprotein, which plays an important role in viral RNA synthesis. ORF3a, ORF6, ORF7a, ORF8, and ORF10 are accessory proteins. Their functions are not fully characterized (Kim et al., 2020).

Clinically, approximately 80% of laboratory-confirmed cases have moderate symptoms, while the remaining ∼20% have severe disease with dyspnea, acute respiratory distress syndrome and/or multiple organ failure, some of whom require the treatment within intensive care unit (ICU). Prolonged deterioration of lymphopenia and high levels of inflammatory cytokines in plasma were found to be associated with severity of COVID-19 (Liu et al., 2020). Subsets of lymphocytes, including CD8+ T cell, CD4+ T cell, B cell, and NK cell, were all significantly lower in severe COVID-19 patients than the moderate patients (He et al., 2020; Huang et al., 2020; Zheng et al., 2020). A recent study using single-cell RNA sequencing (scRNA-seq) of bronchoalveolar lavage fluid (BALF) samples revealed that BALFs from patients with severe symptoms had more proinflammatory monocyte-derived macrophages, higher levels of inflammatory cytokines (IL8, IL6, and IL1B), and less clonally expanded CD8+ T cells than that from the moderate cases, suggesting that cytokine storm is associated with severity of COVID-19 (Liao et al., 2020). However, molecular mechanisms underlying these different immune microenvironment changes between moderate and severe COVID-19 cases remain uncharacterized. We made use of these scRNA-seq datasets together with bulk RNA-seq datasets obtained from BALFs of COVID-19 patients recently published by others (Liao et al., 2020; Xiong et al., 2020; Zhou et al., 2020) to explore types of host cells infected by SARS-CoV-2 and levels of viral gene expression in infected cells. We observed that SARS-CoV-2 sequences were detected in 8 types of immune related cells. Interestingly, we found viral ORF10 was abundantly expressed in the samples from the severe cases but barely detectable in the samples from the moderate cases. Moreover, TRS leader sequence-independent fusion sequences with a 5’ joint point at position 1073 of SARS-CoV-2 genome were detected only in the patients with death outcome. These results implicated that selective expressions of some viral transcripts in infected cells might play roles in severity of COVID-19 and, if so, it might be used as markers for predicting COVID-19 progressions and severity. Our splicing and TRS motif features will help understand how SARS-CoV has evolved at the expression level. We expected more data will be generated in the near future, which will be used to further validate these findings.

## Materials and Methods

### Transcriptome Data

Single-cell RNA sequencing datasets were retrieved from NCBI GEO database (ID: GSE145926^1^). The detailed sequence, demographic (all patients were from Shenzhen, China), and clinical information was provided in Supplementary Tables S1, S2 in the original study (Liao et al., 2020). In this study, we mainly compared sequence and expression features in the infected vs. uninfected cells from severe cases (*n* = 6) and moderate cases (*n* = 3), so the data from normal cases (*n* = 3) were not used due to lack of infected cells. For all the scRNA-seq samples used in this work, 5′ reagent kits were used for library construction. The bulk RNA-seq datasets were obtained from GEO (ID: GSE150316) and the BIGD database (IDs: CRA002423 and CRA002390^2^).

### scRNA-Seq Data Analysis

For analyzing scRNA-seq data, raw reads from scRNA-seq libraries were first cleared by removing the template switch oligo sequence. Reads with adaptor contaminants and low-quality bases were removed. The SARS-CoV-2 reference genome was downloaded from NCBI (MN908947.3, Wuhan-Hu-1 complete genome) including its mapping information of the 10 genes. Alignment of sequence was performed by Cell Ranger software (v. 3.1.0)^3^. Only the reads that were confidently mapped to the SARS-CoV-2 reference genome were used for unique molecular identifier (UMI) counting. We applied UMI > 2 as the cutoff for identification of the infected cells. As a result, distinct UMIs of each gene for a given cell were counted as the transcript copy number of that gene. To reduce the gene expression matrix to its most important features, we used the Uniform Manifold Approximation and Projection (UMAP) method (McInnes et al., 2018) implemented in the Seurat R package and then iteratively clustering strategy to perform subsequent cell clustering. Furthermore, we used Seurat (Stuart et al., 2019) to identify the marker genes of cell groups by calling differentially expressed genes (DEGs) between the cells of each sub-group and the remaining cells. DEGs were selected if the fold change of log2-transformed expression level was > 1 and *p* < 0.05 (Xu et al., 2012; Hua et al., 2020).

### Bulk RNA-Seq Data Analysis

For analysis of bulk RNA-seq data, raw reads were pre-processed using Cutadapt (v1.15) to remove bases with quality scores < 20 and adapter sequences (Martin, 2011). STAR (v2.7.3a) (Dobin et al., 2013) was used for alignment. Then, Stringtie (2.1.2) was used to normalize the gene expression of SARS-CoV-2. We calculated TPM (Transcripts Per Kilobase Million) for gene expression comparison. All statistical tests in this work were performed based on unpaired *t*-test.

## Results

### Infected Cells in Bronchoalveolar Lavage Fluids of COVID-19 Patients

**FIGURE 1:**
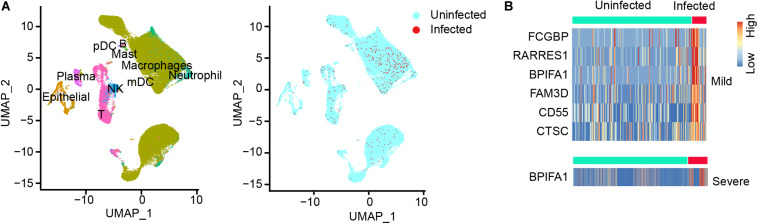
SARS-CoV-2 infected cells and differential gene expression of infected vs. uninfected epithelial cells in BALF samples. **(A)** UMAP (Uniform Manifold Approximation and Projection) view of the cell types for all SARS-CoV-2 infected BALF samples. Color labels each cell cluster identified by scRNA-seq data analysis (see “Materials and Methods”). **(B)** The heatmap of differentially expressed genes (DEGs) of infected epithelial cells vs. uninfected epithelial cells in moderate (mild) cases and in severe cases. One gene, *BPIFA1*, was observed in both moderate and severe cases.

To explore host cells infected by SARS-CoV-2 and viral gene expression in the infected cells, we downloaded public scRNA-seq datasets of bronchoalveolar lavage fluid (BALF) samples obtained from SARS-CoV-2 infected patients, including 3 moderate patients and 6 severe patients (Supplementary Table 1; Liao et al., 2020). The number of total reads ranged from 570,603,931 to 1,417,728,004. The transcriptomic reads of these 9 samples were aligned to SARS-CoV-2 reference genome to detect infected cells. We used UMI > 2 as cutoff for the infected cells. Using the cell type annotations from Liao et al. (2020), we generated UMAP presentation of major cell types in moderate and severe samples, including information of the infected cell and distribution across these cell types (Figure 1A). Our analysis detected 10 cell types. Number of cells varied greatly among those cell types: the top three cell types measured by the largest cell counts were macrophages, T, and epithelia. Among the three moderate cases, only one cell type had measurable infected cells (macrophages) and two cell types had only one infected cell in one or two cases (epithelial and NK). Among the six severe cases, we detected eight cell types with infected cells, though number of infected cells varied widely among those cases (Supplementary Table 2). For neutrophils (including both infected and uninfected neutrophils), we detected an average of 267.2 cells in the samples of severe cases, which is 200-fold more than that (an average of 1.3 cells) in the samples of moderate cases. For epithelial cells (including both infected and uninfected epithelial cells), there were 312.3 cells on average in the samples of severe cases vs. 71.7 cells on average in the samples of moderate cases, a 4.36-fold difference (*t*-test, *p* = 7.71 × 10^–5^) (Supplementary Table 2). The result suggested the presence of severe inflammation and desquamation of lung epithelial cells in the patients with severe COVID-19. We found that the macrophages were highly infiltrated in BALF samples, accounting for 70.2% of all the BALF cells (Supplementary Table 2). SARS-CoV-2 reads were detected in macrophages of all the samples, with an average of 1.86% of the infected cells (Table 1). Plasma cells were relatively few in BALFs, accounting for 2.36% of all the cells in BALFs, with one severe case (S4) as an exception for having a high number of plasma cells (*n* = 814) in BALFs (Table 1 and Supplementary Table 2). In addition, SARS-CoV-2 reads were detected in natural killer (NK) cells and T cells, suggesting the viral infections in these cells (Table 1). We found 1.25–25% NK cells were infected in one moderate sample and three severe samples, respectively (Table 1). In addition, 0.4–6.45% of T cells in five of the six severe samples, but none in the moderate samples, had SARS-CoV-2 reads (Table 1). Infected lung epithelial cells varied from 0.65 to 8.97% and were detected in all the samples. This result suggested that, in addition to epithelial cells, the immune cells, such as macrophages, plasma cells, T cells, and NK cells, can also be infected by SARS-CoV-2.

**TABLE 1:** Proportion of SARS-CoV-2 infected cells.

Cell type	Moderate (% of total cells)			Severe (% of total cells)					
	M1	M2	M3	S1	S2	S3	S4	S5	S6
B	0	0	0	0	0	0	0	0	0
Epithelial	7.23	1.06	2.63	0.65	6.43	2.16	4.59	3.26	8.97
Macrophages	0.55	0.34	1.72	0.17	0.46	1.41	0.61	0.74	10.71
Mast	0	0	0	0	0	0	0	0	0
mDC	0	0	0	0	0	0	0	0	16.67
Neutrophil	0	0	0	0	0	0	0	0.24	2.33
NK	0	0	3.45	0	0	1.25	2.38	0	25.00
pDC	0	0	0	0	0	0	0	2.38	0
Plasma	0	0	0	0	0	0	2.21	0	0
T	0	0	0	0.04	0	0.16	0.38	0.57	6.45

### Differentially Expressed Cellular Genes Between Infected and Uninfected Cells

We called differentially expressed cellular genes in the infected vs. uninfected cells in each cell type. The number of cells in each sub-group is summarized in Supplementary Table 2. Using the cutoff of fold change > 2 and *p* < 0.05, we discovered 6 DEGs (*FCGBP, RARRES1, BPIFA1, FAM3D, CD55*, and *CTSC*) which were highly expressed in infected vs. uninfected lung epithelial cells of moderate patients (Figure 1B). However, *BPIFA1* was the only gene found highly expressed in the infected vs. uninfected lung epithelial cells of the severe patients (Figure 1B). *BPIFA1* encodes a secretory protein and is known to have antimicrobial activity. A recent study showed that *BPIFA1* regulates lung neutrophil infiltration and interferon signaling during acute inflammation (Britto et al., 2019). Its overexpression may play a role in inflammatory response of SARS-CoV-2 infection in lung. Among the other five genes, *FCGBP*, which encodes Fc fragment of IgG binding protein, has been reported with virus infection and viral vector design. *RARRES1* encodes retinoic acid receptor responder 1. It is involved in negative regulation of cell proliferation and endopeptidase activity. *FAM3D*, which encodes FAM3 metabolism regulating signaling molecule D, has Gene Ontology annotations related to cytokine activity. *CD55* encodes a glycoprotein involved in the regulation of the complement cascade. *CTSC* a member of the peptidase C1 family and lysosomal cysteine proteinase. It involves in the activation of many serine proteinases in cells of the immune system.

### SARS-CoV-2 Gene Expression in Severe and Moderate Cases

**FIGURE 2:**
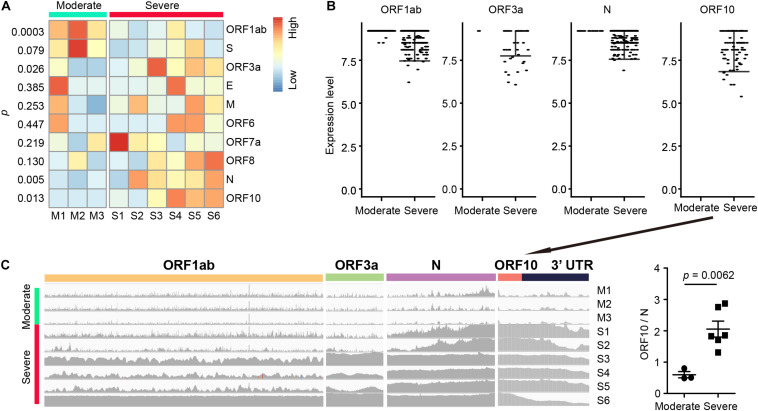
SARS-CoV-2 gene expression and RNA fusions in BALF samples. **(A)** Differential SARS-CoV-2 gene expression (measured by normalized average gene expression) between the moderate and severe groups. **(B)** Expression of four SARS-CoV-2 genes (ORF1ab, ORF3a, N, and ORF10) in each infected cell. Each dot represents an infected cell. **(C)** Levels of read coverages in different viral regions in each sample (left) and ratio of coverage of ORF10 vs. N (right, ORF10/N). The unpaired t test was used for all the statistical tests.

To detect differentially expressed viral genes, we compared the normalized expression of SARS-CoV-2 genes between severe and moderate patients (Figure 2A). With the cutoff *p* < 0.05, we found 4 genes (ORF1ab, ORF3a, N, and ORF10) differentially expressed in severe vs. moderate scRNA-seq samples. Interestingly, ORF10 was only detected in the infected cells of severe case samples (Figure 2B). All samples from severe cases had a high sequence coverage for ORF10. In contrast, ORF10 was rarely detected in the infected cells of BALF from moderate cases (Figure 2C). We compared the ratio of sequence coverage of ORF10 to that of N. This ratio was significantly higher in severe than moderate samples (3.43-folds, *p* = 0.0062, Figure 2C). This result suggested that differential expression of ORF10 may be a potential biomarker for COVID-19 progression.

### SARS-CoV-2 Genomic Fusions in Host Cells

**FIGURE 3:**
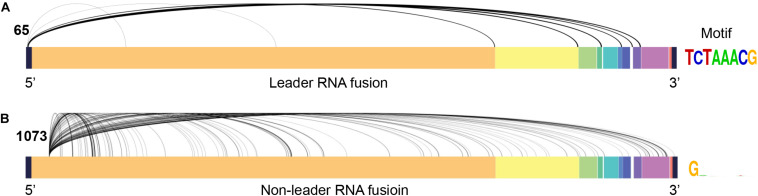
Major viral RNA fusions detected in infected cells. **(A)** TRS-L dependent RNA fusions. **(B)** The most frequent TRS-L independent RNA fusions with a 5’ position at 1073 in SARS-CoV-2 genome.

We observed several viral RNA fusion events (Supplementary Table 3) in the scRNA-seq datasets, as well as in bulk RNA-seq datasets from Xiong et al. (2020) and Zhou et al. (2020) (Supplementary Table 1). For the transcription regulatory sequence of the viral leader sequence (TRS-L) dependent fusion groups started at position 65, we found a strong signal motif (TCTAAACG) (Figure 3A and Supplementary Table 4). In addition, we found a highly frequent TRS-L independent fusions that occurred at 5′ position 1073, which fused to multiple 3′ positions without the consistent splicing motif at 3′ locations (Figure 3B and Supplementary Table 5). TRS-L dependent fusion transcripts are known for the classical sgRNA being formed with a heptameric template-switching signal motif (TCTAAAC), which is critical for viral gene expressions in coronavirus, as reported before COVID-19 outbreak (Wu and Brian, 2010). Here, we reported a very similar, but one nucleotide longer, motif in SARS-CoV-2 genomes (TCTAAACG in SARS-CoV-2 vs. TCTAAAC in SARS-CoV). By manually checking the reads being mapped to TRS-L independent fusions started at 1073 one by one in our data, we found that 14 patients had high-quality reads being mapped to 1,073 (Table 2), and 5 out of the 14 patients had the 5’ fusion reads at this position. The clinical and sequence information of these 14 patients is provided in Supplementary Table 1. Remarkably, all these 5 patients unfortunately passed away (Table 2), suggesting that presence of fusions with a 5′ position at 1073 might be associated with worse outcome of COVID-19 patients.

**TABLE 2:** Summary of fusion reads starting from SARS-CoV-2 position 1073 in each patient.

Sample name	Type	# Fusion reads	# Total reads	Proportion (%)	Clinical outcome	Source
S3	scRNA-seq	0	255	0	Cured	BALF
S4	scRNA-seq	0	18	0	Cured	BALF
S5	scRNA-seq	1	8	12.50	Death	BALF
S6	scRNA-seq	23	410	7.80	Death	BALF
Case1	Bulk RNA-seq	233	460	50.65	Death	Autopsy
Case2	Bulk RNA-seq	1	1	100	Death	Autopsy
Case5	Bulk RMA-seq	6	6	100	Death	Autopsy
WIV02	Bulk RNA-seq	0	2	0	Cured	BALF
WIV04	Bulk RNA-seq	0	13	0	Cured	BALF
WIV05	Bulk RNA-seq	0	1	0	Cured	BALF
WIV06	Bulk RNA-seq	0	5	0	Cured	BALF
WIV07	Bulk RNA-seq	0	16	0	Cured	BALF
Patient_1	Bulk RNA-seq	0	550	0	Cured	BALF
Patient_2	Bulk RNA-seq	0	193	0	Cured	BALF

*The clinical and sequence information of these 14 patients is provided in Supplementary Table 1 (both scRNA-seq and bulk RNA-seq data).*

## Discussion

Our results revealed that both epithelial cells and immune cells were infected with the SARS-CoV-2. These immune cells included macrophages, monocyte dendritic cells (mDCs), plasmacytoid dendritic cells (pDCs), neutrophils, natural killers (NK), plasma cells, and T cells. The infected epithelial cells and macrophages were found in all moderate and severe cases, indicating that the SARS-CoV-2 virulence could be involved in immune and the related cells. The air way and lung epithelial cells serve important functions as barrier protection for respiratory tract. SARS-CoV-2 is known to infect lung epithelial cells through membrane fusion mediated by binding of SARS-CoV-2’s spike to ACE2 on cell surface (Sungnak et al., 2020; Ziegler et al., 2020). Macrophages are the key phagocytes which can take up pathogens. Our results showed that macrophages in all COVID-19 cases were detectable by SARS-CoV-2 sequences. The infected T cells were only found in the severe cases. Middle East respiratory syndrome (MERS) coronavirus can infect T lymphocytes and induce T-cell apoptosis through extrinsic and intrinsic apoptosis pathways (Chu et al., 2016). Whether SARS-CoV-2 also induces T cell apoptosis remains to be determined. *BPIFA1* was highly expressed in both moderate and severe infected epithelial cells, which may play an important role in regulating lung neutrophil infiltration interferon signaling during acute inflammation (Britto et al., 2019).

By comparing SARS-CoV-2 mapped reads between severe and moderate cases, we found that ORF10 was barely expressed in the moderate cases, but it could be detected in high confidence in the severe cases. The ratio of the short-read sequence coverage of ORF10 to that of N in the severe cases was 3.43-fold higher than that in the moderate cases (*p* = 0.0062, Figure 2C). The biological significance of the lack of ORF10 expression in infected cells of moderate cases is not yet clear. A recent study on SARS-CoV-2 sgRNAs in viral replication and gene expression in Vero cells suggested that ORF10 might not be expressed as a protein (Kim et al., 2020). Since ORF10 is located close to 3’ untranslated region (UTR), it is possible that ORF10 may play a role in regulating transcription or stability of viral sgRNAs. Some previous studies indicated that single-strand virus RNA decays at 3’ UTR through nonsense-mediated decay (Balistreri et al., 2014; May et al., 2018). Because only one time point is used for moderate and severe cases in this study, it is not clear whether the difference of ORF10 coverage between moderate and severe cases was related to the decay of SARS-CoV-2’s sgRNA and/or gRNA during the disease course. By further exploration of the SARS-CoV-2 reads, we found numerous SARS-CoV-2 RNA genome fusion events in the host cells, suggesting that splicing may be a critical evolutionary mechanism for SARS-CoV-2 to adapt the host cells. Most of them belonged to transcription regulatory sequence of the viral leader sequence (TRS-L) dependent fusion groups, with a strong motif (TCTAAACG). Among TRS-L independent fusions, we found one fusion with a 5’ position at 1073. This fusion is most common when compared to other TRS-L independent fusions, and it is only detected in samples of dead cases. Our finding suggested that the presence of these fusions could be associated with worse outcome of COVID-19 patients.

In summary, through re-analysis of the datasets from a recently published scRNA-seq of BALF samples from 9 COVID-19 patients (3 moderate and 6 severe symptoms), we reported that viral ORF10 was highly expressed in severe cases compared to moderate cases. We also reported that the presence of TRS leader sequence-independent fusion sequences with a 5’ joint point at position 1073 of SARS-CoV-2 genome was associated with the worst outcome of COVID-19 patients. These results implicated that selective expressions of some viral transcripts might help strengthen virulence If so, such a selective expression might play roles in pathogenesis of COVID-19 and might be used as markers for predicting disease progression and death outcome. Nevertheless, there are several limitations in our results. First, the sample size is very small – we used the scRNA-seq data of bronchoalveolar lavage fluid samples from only 3 moderate patients and 6 severe patients to explore SARS-CoV-2 viral gene expression in the infected cells. Due to the difficulty in obtaining such tissues from COVID-19 patients, this unique dataset is currently what we could find in the field. Second, while the demographic background is consistent (all the samples were collected from Shenzhen, China), age, pre-existing conditions and other clinical factors could not be fully addressed due to small sample size, as discussed in the original study (Liao et al., 2020). The age in the severe case groups was older than that in the moderate case groups, which is consistent with the general clinical reports of older patients having high risk to COVID-19 severity. Third, because the sample size is very small, longitudinal data were not available, and disease progression only covered two stages (moderate and severe; though all the death outcome of the bulk RNA-seq samples in Zhou et al. (2020) and Xiong et al. (2020) studies was confirmed through personal communications.). It remains unclear whether the observed difference was caused by dynamic changes in different time course of SARS-CoV-2 infection in patients. Further validation studies are required before ORF10 expression or presence of 1073 fusions can be used to predict disease progression for COVID-19. As more SARS-CoV-2 genomes will be generated from infected samples in the near future, we will validate our findings when such data are released.

## Data Availability Statement

The raw data supporting the conclusions of this article can be downloaded from NCBI GEO database (IDs: GSE145926 and GSE150316, https://www.ncbi.nlm.nih.gov/geo/) and the BIGD database (IDs: CRA002423 and CRA002390, https://bigd.big.ac.cn/). Analyzed results will be made available by the authors, without undue reservation, to any qualified researcher. Requests to access these datasets should be directed to GEO and BIGD.

## Author Contributions

TL and ZZ conceived the study. TL, PJ, BF, and ZZ performed the research and wrote the manuscript. TL collected and analyzed the data. All authors contributed to the article and approved the manuscript.

## Conflict of Interest

The authors declare that the research was conducted in the absence of any commercial or financial relationships that could be construed as a potential conflict of interest.

## Footnotes

1 https://www.ncbi.nlm.nih.gov/geo/

2 https://bigd.big.ac.cn/

3 https://support.10xgenomics.com/
