# Supplementary material for: Differential Expression of Viral Transcripts From Single-Cell RNA Sequencing of Moderate and Severe COVID-19 Patients and Its Implications for Case Severity
Source: Front Microbiol. 2020 Oct 16;11:603509. doi: 10.3389/fmicb.2020.603509 (PMC7596306; doi:10.3389/fmicb.2020.603509)
Supplement: Supplementary file 1 [file Table_1.DOCX]

**Supplementary Table 1.** Summary of SARS-CoV-2 alignment from scRNA-seq and bulk RNA-seq data.

| Sample ID | Sample type | Sample name | Total reads | Mapped reads to SARS-CoV-2 | Description | Sample acquisition date | Health status | Clinical  outcome |
| --- | --- | --- | --- | --- | --- | --- | --- | --- |
| SRR11181954 | scRNA-seq | M1 | 1412724793 | 84571 | BALF | 2020/1/20 | Mild | Cured |
| SRR11181955 | scRNA-seq | M2 | 1232446428 | 142189 | BALF | 2020/1/20 | Mild | Cured |
| SRR11181957 | scRNA-seq | M3 | 1238159803 | 121928 | BALF | 2020/1/22 | Mild | Cured |
| SRR11181956 | scRNA-seq | S1 | 1417728004 | 41687 | BALF | 2020/1/22 | Severe | Cured |
| SRR11537949 | scRNA-seq | S2 | 656828360 | 18918 | BALF | 2020/1/21 | Severe | Cured |
| SRR11537950 | scRNA-seq | S3 | 570603931 | 109367 | BALF | 2020/1/22 | Severe | Cured |
| SRR11537951 | scRNA-seq | S4 | 646965801 | 71222 | BALF | 2020/1/29 | Severe | Cured |
| SRR11181958 | scRNA-seq | S5 | 1309990564 | 102481 | BALF | 2020/1/29 | Severe | Dead |
| SRR11181959 | scRNA-seq | S6 | 1252013349 | 1551847 | BALF | 2020/1/22 | Severe | Dead |
| CRR119894 | Bulk RNA-seq | Patient 1 repeat 1 | 16893416 | 273414 | BALF |  | Severe | Cured |
| CRR119895 | Bulk RNA-seq | Patient 1 repeat 2 | 7369998 | 86414 | BALF |  | Severe | Cured |
| CRR119896 | Bulk RNA-seq | Patient 2 repeat 1 | 9530602 | 64290 | BALF |  | Severe | Cured |
| CRR119897 | Bulk RNA-seq | Patient 2 repeat 2 | 4522806 | 22594 | BALF |  | Severe | Cured |
| CRR122276 | Bulk RNA-seq | WIV02 | 16534058 | 418 | BALF | 2019/12/27 | Severe | Cured |
| CRR122277 | Bulk RNA-seq | WIV04 | 10369826 | 3360 | BALF | 2019/12/29 | Severe | Cured |
| CRR122278 | Bulk RNA-seq | WIV06 | 10479446 | 142 | BALF | 2019/12/29 | Severe | Cured |
| CRR122279 | Bulk RNA-seq | WIV07 | 7133168 | 732 | BALF | 2019/12/20 | Severe | Cured |
| CRR122280 | Bulk RNA-seq | WIV02Rep1 | 56769552 | 820 | BALF | 2019/12/27 | Severe | Cured |
| CRR122281 | Bulk RNA-seq | WIV02Rep2 | 77396838 | 1546 | BALF | 2019/12/27 | Severe | Cured |
| CRR122282 | Bulk RNA-seq | WIV04Rep1 | 69951796 | 11374 | BALF | 2019/12/29 | Severe | Cured |
| CRR122283 | Bulk RNA-seq | WIV04Rep2 | 52656314 | 19228 | BALF | 2019/12/29 | Severe | Cured |
| CRR122284 | Bulk RNA-seq | WIV05 | 68511686 | 1232 | BALF | 2019/12/29 | Severe | Cured |
| CRR122285 | Bulk RNA-seq | WIV06Rep1 | 59351670 | 2294 | BALF | 2019/12/28 | Severe | Cured |
| CRR122286 | Bulk RNA-seq | WIV07Rep1 | 76928388 | 4952 | BALF | 2019/12/20 | Severe | Cured |
| SRR11772358 | Bulk RNA-seq | Case1 | 51545938 | 523806 | Case1-lung1 |  | Autopsy | Dead |
| SRR11772359 | Bulk RNA-seq | Case1 | 7029892 | 55403 | Case1-lung2 |  | Autopsy | Dead |
| SRR11772360 | Bulk RNA-seq | Case1 | 5572880 | 21066 | Case1-lung3 |  | Autopsy | Dead |
| SRR11772361 | Bulk RNA-seq | Case1 | 3123330 | 37808 | Case1-lung4 |  | Autopsy | Dead |
| SRR11772363 | Bulk RNA-seq | Case2 | 7395998 | 488 | Case2-lung1 |  | Autopsy | Dead |
| SRR11772364 | Bulk RNA-seq | Case2 | 8383638 | 42 | Case2-lung2 |  | Autopsy | Dead |
| SRR11772366 | Bulk RNA-seq | Case2 | 8219322 | 14 | Case2-lung3 |  | Autopsy | Dead |
| SRR11772368 | Bulk RNA-seq | Case3 | 49775474 | 44 | Case3-lung1 |  | Autopsy | Dead |
| SRR11772370 | Bulk RNA-seq | Case3 | 15191290 | 12 | Case3-lung2 |  | Autopsy | Dead |
| SRR11772371 | Bulk RNA-seq | Case4 | 13642346 | 46 | Case4-lung1 |  | Autopsy | Dead |
| SRR11772374 | Bulk RNA-seq | Case4 | 12003810 | 48 | Case4-lung2 |  | Autopsy | Dead |
| SRR11772378 | Bulk RNA-seq | Case5 | 13837040 | 242 | Case5-lung1 |  | Autopsy | Dead |
| SRR11772379 | Bulk RNA-seq | Case5 | 11400442 | 78 | Case5-lung2 |  | Autopsy | Dead |
| SRR11772380 | Bulk RNA-seq | Case5 | 14612014 | 660 | Case5-lung3 |  | Autopsy | Dead |
| SRR11772381 | Bulk RNA-seq | Case5 | 16156442 | 522 | Case5-lung4 |  | Autopsy | Dead |
| SRR11772383 | Bulk RNA-seq | Case5 | 15833118 | 748 | Case5-lung5 |  | Autopsy | Dead |

BALF: Bronchoalveolar lavage fluids.
