# Supplementary material for: Differential Expression of Viral Transcripts From Single-Cell RNA Sequencing of Moderate and Severe COVID-19 Patients and Its Implications for Case Severity
Source: Front Microbiol. 2020 Oct 16;11:603509. doi: 10.3389/fmicb.2020.603509 (PMC7596306; doi:10.3389/fmicb.2020.603509)
Supplement: Supplementary file 2 [file Table_2.DOCX]

**Supplementary Table 2.** Counts of the cells and infected cells (in parentheses) by cell type.

| Cell type | M | | |  | S | | | | | |
| --- | --- | --- | --- | --- | --- | --- | --- | --- | --- | --- |
|  | M1 | M2 | M3 |  | S1 | S2 | S3 | S4 | S5 | S6 |
| B | 35 (0) | 24 (0) | 8 (0) |  | 28 (0) | 4 (0) | 28 (0) | 6 (0) | 34 (0) | 2 (0) |
| Epithelial | 83 (6) | 94 (1) | 38 (1) |  | 309 (2) | 373 (24) | 185 (4) | 327 (15) | 368 (12) | 312 (28) |
| Macrophages | 2177 (12) | 2060 (7) | 116 (2) |  | 12765 (22) | 1087 (5) | 1062 (15) | 1147 (7) | 9829 (73) | 635 (68) |
| Mast | 2 (0) | 2 (0) | 2 (0) |  | 13 (0) | 6 (0) | 8 (0) | 4 (0) | 8 (0) | 0 (0) |
| mDC | 85 (0) | 55 (0) | 19 (0) |  | 116 (0) | 25 (0) | 14 (0) | 6 (0) | 161 (0) | 12 (2) |
| Neutrophil | 2 (0) | 1 (0) | 1 (0) |  | 829 (0) | 31 (0) | 50 (0) | 24 (0) | 412 (1) | 257 (6) |
| NK | 130 (0) | 139 (0) | 29 (1) |  | 357 (0) | 21 (0) | 80 (1) | 42 (1) | 178 (0) | 8 (2) |
| pDC | 33 (0) | 24 (0) | 3 (0) |  | 32 (0) | 1 (0) | 0 (0) | 1 (0) | 42 (1) | 0 (0) |
| Plasma | 11 (0) | 2 (0) | 1 (0) |  | 162 (0) | 13 (0) | 4 (0) | 814 (18) | 27 (0) | 4 (0) |
| T | 981 (0) | 1,008 (0) | 146 (0) |  | 2,272 (1) | 155 (0) | 638 (1) | 526 (2) | 703 (4) | 62 (4) |
