# Supplementary material for: Differential Expression of Viral Transcripts From Single-Cell RNA Sequencing of Moderate and Severe COVID-19 Patients and Its Implications for Case Severity
Source: Front Microbiol. 2020 Oct 16;11:603509. doi: 10.3389/fmicb.2020.603509 (PMC7596306; doi:10.3389/fmicb.2020.603509)
Supplement: Supplementary file 3 [file Table_3.DOCX]

**Supplementary Table 3.** Top ten 5’ fusion locations and supported reads.

| 5’ location (nt) | Sample count | # support reads |
| --- | --- | --- |
| 65 | 12 | 260 |
| 1073 | 5 | 256 |
| 4132 | 4 | 41 |
| 27799 | 5 | 34 |
| 4957 | 4 | 20 |
| 12471 | 5 | 14 |
| 83 | 5 | 14 |
| 22271 | 5 | 11 |
| 8246 | 4 | 9 |
| 3989 | 4 | 6 |
