# Supplementary material for: Differential Expression of Viral Transcripts From Single-Cell RNA Sequencing of Moderate and Severe COVID-19 Patients and Its Implications for Case Severity
Source: Front Microbiol. 2020 Oct 16;11:603509. doi: 10.3389/fmicb.2020.603509 (PMC7596306; doi:10.3389/fmicb.2020.603509)
Supplement: Supplementary file 4 [file Table_4.DOCX]

**Supplementary Table 4.** Summary of 5’, 3’, and patients of leader RNA fusion.

| 5' (nt) | 3' (nt) | # patients |
| --- | --- | --- |
| 65 | 28255 | 8 |
| 65 | 25381 | 7 |
| 65 | 26468 | 7 |
| 65 | 27884 | 6 |
| 65 | 27385 | 5 |
| 65 | 21552 | 4 |
| 65 | 11488 | 1 |
| 65 | 27384 | 1 |
| 65 | 4585 | 1 |
