# Supplementary material for: Differential Expression of Viral Transcripts From Single-Cell RNA Sequencing of Moderate and Severe COVID-19 Patients and Its Implications for Case Severity
Source: Front Microbiol. 2020 Oct 16;11:603509. doi: 10.3389/fmicb.2020.603509 (PMC7596306; doi:10.3389/fmicb.2020.603509)
Supplement: Supplementary file 5 [file Table_5.DOCX]

**Supplementary Table 5.** Summary of 5’, 3’, and patients of the most frequent non-leader RNA fusion.

| 5' (nt) | 3' (nt) | # patients | Peptides |
| --- | --- | --- | --- |
| 1073 | 29758 | 1 | GGVYSEQC |
| 1073 | 29435 | 2 | GEETANCD |
| 1073 | 29426 | 1 | GAETEETA |
| 1073 | 29399 | 2 | GEG--NSS |
| 1073 | 29396 | 3 | GEEG--NS |
| 1073 | 29244 | 1 | GEVTPSGT |
| 1073 | 29045 | 2 | GEASAKTY |
| 1073 | 29018 | 1 | GEEICC-G |
| 1073 | 28797 | 1 | GEGSRGGS |
| 1073 | 28629 | 2 | GEAGLPYG |
| 1073 | 28540 | 1 | GEELPDEF |
| 1073 | 28461 | 1 | GEDLKFPR |
| 1073 | 28213 | 1 | GEDFLEYH |
| 1073 | 27671 | 1 | GEVQELYS |
| 1073 | 27379 | 1 | GGD-LNEH |
| 1073 | 26557 | 1 | GEELKKLL |
| 1073 | 26269 | 1 | GEDRYVNS |
| 1073 | 26267 | 1 | GEETGTLI |
| 1073 | 26117 | 1 | GEEHVQIH |
| 1073 | 25700 | 2 | GEAPFLYL |
| 1073 | 25441 | 1 | GEAR-NQG |
| 1073 | 25365 | 1 | GGVKLHYT |
| 1073 | 25338 | 1 | GEDDSEPV |
| 1073 | 24840 | 1 | GEGVFVSN |
| 1073 | 24021 | 1 | GEDLLFNK |
| 1073 | 23904 | 1 | GEVFAQVK |
| 1073 | 23421 | 1 | GEVPVAIH |
| 1073 | 23016 | 1 | GEGFNCYF |
| 1073 | 22786 | 1 | GADKSLQG |
| 1073 | 22782 | 1 | GEVRQIAP |
| 1073 | 22303 | 1 | GEAI-LLV |
| 1073 | 21854 | 2 | GEV-HNKR |
| 1073 | 21452 | 1 | GEGQINDM |
| 1073 | 21269 | 1 | GEAFLIGC |
| 1073 | 20420 | 1 | GEDFIPMD |
| 1073 | 20306 | 1 | GEGYAFEH |
| 1073 | 20057 | 1 | GEGSVKGL |
| 1073 | 18969 | 1 | GEERFNTW |
| 1073 | 18952 | 1 | GED-CGL- |
| 1073 | 18628 | 1 | GEVFWKNR |
| 1073 | 18272 | 1 | GEEAIRHV |
| 1073 | 18149 | 1 | GEGL-VDI |
| 1073 | 16706 | 1 | GEVLSDRE |
| 1073 | 14739 | 1 | GEVLLN-N |
| 1073 | 13691 | 2 | GEETIYNL |
| 1073 | 13625 | 1 | GEDDNLID |
| 1073 | 13044 | 1 | GEVPANST |
| 1073 | 12944 | 1 | GEVFILY- |
| 1073 | 12212 | 2 | GEVFECG- |
| 1073 | 12209 | 1 | GEEVFECG |
| 1073 | 12189 | 1 | GEVVLKKV |
| 1073 | 12153 | 2 | GEAYEQAV |
| 1073 | 12063 | 1 | GEEMLNNR |
| 1073 | 11985 | 1 | GEAFEKMV |
| 1073 | 10626 | 1 | GNSTQLVR |
| 1073 | 10590 | 1 | GEGNFYGP |
| 1073 | 10197 | 1 | GEDMLNPN |
| 1073 | 9951 | 1 | GEAASCHL |
| 1073 | 9798 | 1 | GEAALFPF |
| 1073 | 9795 | 1 | GEEAALCT |
| 1073 | 9246 | 1 | GEAGVCVS |
| 1073 | 9177 | 1 | GEGSVRVV |
| 1073 | 9087 | 1 | GEGSVAYE |
| 1073 | 8850 | 1 | GEEGFVVP |
| 1073 | 8259 | 1 | GEVTGDSC |
| 1073 | 8118 | 1 | GEAELAKN |
| 1073 | 7917 | 1 | GEESSAKS |
| 1073 | 7757 | 1 | GEEWFHPS |
| 1073 | 7671 | 1 | GEVARDLS |
| 1073 | 6417 | 1 | GEVVENPT |
| 1073 | 6414 | 1 | GEEVVENP |
| 1073 | 5565 | 1 | GEAVMYMG |
| 1073 | 5385 | 1 | GEGKTFYV |
| 1073 | 5109 | 1 | GEEHFIET |
| 1073 | 4761 | 1 | GEAARYMR |
| 1073 | 4650 | 1 | GEEAARYM |
| 1073 | 4647 | 1 | GEETRKLM |
| 1073 | 4416 | 1 | GEETKFLT |
| 1073 | 3999 | 1 | GEVTTTLE |
| 1073 | 3981 | 1 | GEEVTTTL |
| 1073 | 3978 | 1 | GEVKPFIT |
| 1073 | 3897 | 1 | GEE-KAS- |
| 1073 | 3848 | 1 | GEVLLAPL |
| 1073 | 3693 | 1 | GEAKTNSG |
| 1073 | 3419 | 1 | GEAKKVKP |
| 1073 | 3411 | 1 | GEEAKKVK |
| 1073 | 3408 | 2 | GEVNSFSG |
| 1073 | 3339 | 1 | GEDWLDDD |
| 1073 | 3198 | 1 | GEEDWLDD |
| 1073 | 3195 | 2 | GEEQEEDW |
| 1073 | 3186 | 2 | GEEQEENW |
| 1073 | 3183 | 1 | GEEEQEED |
| 1073 | 3123 | 1 | GEDDYQGK |
| 1073 | 3084 | 1 | GEEFEPST |
| 1073 | 3081 | 1 | GEEEFEPS |
| 1073 | 3078 | 1 | GEEEEFEP |
| 1073 | 3066 | 1 | GEGDCEEE |
| 1073 | 3063 | 1 | GEEGDCEE |
| 1073 | 3060 | 1 | GEEEGDCE |
| 1073 | 2868 | 1 | GEVNEFAC |
| 1073 | 2834 | 1 | GEVLCLYS |
| 1073 | 2763 | 1 | GEVQGYKS |
| 1073 | 2592 | 1 | GEAPLVGT |
| 1073 | 2532 | 1 | GEVVLKTG |
| 1073 | 2442 | 1 | GEETGLLM |
| 1073 | 2178 | 1 | GEGVEFLR |
| 1073 | 2168 | 1 | GEV-GRCR |
| 1073 | 2163 | 1 | GEEKFKEG |
| 1073 | 1680 | 1 | GEEIAIIL |
| 1073 | 1599 | 1 | GEGLNDNL |
| 1073 | 1590 | 1 | GEGSEGLN |
